# Innovations in nutrition education and global health: the Bangalore Boston nutrition collaborative

**DOI:** 10.1186/1472-6920-14-5

**Published:** 2014-01-08

**Authors:** Rebecca Kuriyan, Jeffrey K Griffiths, Julia L Finkelstein, Tinku Thomas, Tony Raj, Ronald J Bosch, Anura V Kurpad, Christopher Duggan

**Affiliations:** 1 Division of Nutrition, St. John’s Research Institute, St. John’s National Academy of Health Sciences, Sarjapur Road, Bangalore 560034, INDIA; 2 Department of Public Health and Community Medicine, Tufts University School of Medicine, Boston, MA, USA; 3 Division of Nutritional Sciences, Cornell University, Ithaca, NY, USA; 4 Department of Biostatistics, Harvard School of Public Health, Boston, MA, USA; 5 Division of GI/Nutrition, Boston Children’s Hospital, and Department of Nutrition, Harvard School of Public Health, Boston, MA, USA

**Keywords:** Nutrition, Global Health, Education, Training, Research

## Abstract

**Background:**

India has a wide range of nutrition and health problems which require professionals with appropriate skills, knowledge and trans-disciplinary collaborative abilities to influence policy making at the national and global level.

**Methods:**

The Bangalore Boston Nutrition Collaborative (BBNC) was established as collaboration between St. John’s Research Institute (SJRI), Harvard School of Public Health and Tufts University, with a focus on nutrition research and training. The goals of the BBNC were to conduct an interdisciplinary course, develop web-based courses and identify promising Indian students and junior faculty for graduate training in Boston.

**Results:**

From 2010, an annual two-week short course in nutrition research methods was conducted on the SJRI campus taught by international faculty from Indian and US universities. More than 100 students applied yearly for approximately 30 positions. The course had didactic lectures in the morning and practical hands-on sessions in the afternoon. Student rating of the course was excellent and consistent across the years. The ratings on the design and conduct of the course significantly improved (p <0.001) from 2010 to 2012. Through open-ended questions, students reported the main strengths of the course to be the excellent faculty and practical “hands-on” sessions. A web based learning system TYRO, was developed, which can be used for distance learning. Four faculty members/graduate students from SJRI have visited Boston for collaborative research efforts.

**Conclusion:**

The BBNC has become a well-established capacity building and research training program for young professionals in nutrition and global health. Efforts are ongoing to secure long term funding to sustain and expand this collaboration to deliver high quality nutrition and global health education enabled by information and communication technologies.

## Background

Nutrition plays a critical role in promoting and maintaining optimal health throughout the life cycle. India faces a dual burden of nutrition-related diseases, with high levels of maternal and child mortality and morbidity directly related to undernutrition, micronutrient deficiencies, and infectious diseases paired with a growing incidence of non-communicable diseases based on western diet and activity patterns [1,2]. Rural Indian children under age 5 years have a high prevalence of stunting (48%), underweight (43%), and wasting (20%), and up to 70% are anemic [3]. Meanwhile, the prevalence of childhood and adolescent overweight and obesity ranges from 3-29% [4]. This double burden of nutrition-related diseases leads to a wide range of health problems that will impede both individual and national economic attainment [5]. Thus, the challenge for India is to implement appropriate prevention strategies in order to halt the growing trend in non-communicable diseases against a background of infectious diseases which still remains highly prevalent.

Critical to these efforts is the development of Indian professionals with appropriate skills, knowledge and abilities to work together across various disciplines to design appropriate research studies that influence policy-making at the local, national and global level [6,7]. Public health education covering the domains of biostatistics, epidemiology, nutritional epidemiology, research ethics and nutritional biochemistry is needed to develop professional competence in the field of nutrition research. Skilled professionals in the fields of epidemiology, biostatistics, social sciences and public health are needed in order to better document the increasing rate of chronic disease epidemics in India and to devise optimal methods for their prevention and treatment. There are about 355 recognized medical colleges in India [8] and 190 institutes that offer nutrition courses at various academic levels such as certificate course, Bachelors (BSc), Masters (MSc), post graduate diploma (PG) and Doctorate (PhD) [9]. However, public health nutrition (PHN) is not available as an independent discipline in any college/university across India, with only 3 colleges offering Diploma programs [9]. PHN education and training programs could help in understanding and addressing the existing dual burden of diseases in India. Thus, there is an immediate need for quality training programs to train faculty to acquire the necessary skills and competencies to conduct high quality research.

With this training gap in mind, the Bangalore Boston Nutrition Collaborative (BBNC) was established in 2009 as a sustainable educational collaboration between nutrition scientists at St. John’s Research Institute in Bangalore (SJRI) and colleagues at Harvard School of Public Health (HSPH) and Tufts University Schools of Medicine and Nutrition in Boston. The main goal of BBNC was to conduct an interdisciplinary course providing substantive knowledge and methodological skills in nutrition research, with applications to clinical, research, program, policy, and laboratory areas. Additional goals were to develop web-based courses in epidemiology, biostatistics, nutritional epidemiology and nutritional biochemistry, and to identify promising Indian students and junior faculty for graduate training or short courses in Boston. We describe the conception, implementation and evaluation of this unique, multi-institutional educational collaboration.

## Methods

### Leadership and planning

The BBNC was funded by a grant from an anonymous Boston-based foundation, and was built on a history of over two decades of collaboration between researchers at HSPH, Tufts University and SJRI. An Executive Committee consisting of faculty and a Research and Curriculum Coordinator was appointed. During the first six months of this initiative (July- December 2009), planning was undertaken for the inaugural course via regular conference calls. We established the Bangalore Boston Nutrition Collaborative website (http://bbnc.globalhealth.harvard.edu), and invited additional faculty members from SJRI, HSPH, and Tufts to teach in the intensive short course in Bangalore. We also developed the course curriculum and materials, contacted institutions in India for prospective students, circulated course advertisements, and planned together for the inauguration of the intensive short course in Bangalore.

### I. International course in nutrition research methods

The BBNC International Course in Nutrition Research Methods is an intensive interdisciplinary two week short course offered annually at St. John’s Research Institute in Bangalore, India. The short course aims to train the students in planning research studies from “Cell to Society”. Since adequate faculty interaction is critical for effective learning and skill acquisition, the BBNC short course was designed with didactic lectures in the morning, followed by interactive, problem-based discussion and hands-on practicums in the afternoon. Since an integrated course in nutrition research methods is lacking in India, the BBNC short course was designed with the following objectives:

(1) To explore the role of nutrition in health outcomes, through critical evaluation of the scientific literature and exploration of demographic, epidemiological, biological, social, political, and economic factors that affect nutritional status.

(2) To foster substantive knowledge in nutrition research, including clinical nutrition, physiology, biochemistry, and molecular nutrition, and the role of nutrition in perinatal health, infectious diseases, and non-communicable diseases.

(3) To transmit methodological skills in nutrition research in the areas of clinical nutrition, epidemiology, biostatistics and research ethics, with emphasis on clinical, research, and laboratory methods.

(4) To examine the latest findings from epidemiologic studies on the role of nutrition in the prevention, care, and treatment of health problems, and inform the development of research studies and nutrition programs, with a emphasis on Indian public health problems.

#### Application process and students

Students for the BBNC short course were selected through a competitive application process. Advertisements for the course were posted on the website of SJRI and communicated to Indian and South Asian academic organizations, research groups, hospitals and public health institutes by email, posters and flyers. Researchers in US universities with collaborative studies in India were also contacted. Candidates completed written applications detailing prior education and training, current position, planned future activities and positions, and submitted two letters of recommendation from current supervisors. Students were selected by a committee composed of Bangalore and Boston faculty based on the following criteria: professional training, commitment to nutrition, main area of research interest and academic potential.

There were no course fees and all participants received complete financial support for travel, accommodations and food expenses. All participants also received a course kit which included a textbook in biostatistics [10] and all course materials, including speaker slides, lecture notes and readings from the literature. Student travel, accommodation and food expenses were supported in part by small training grants from Sight and Life, a humanitarian initiative of Dutch States Mines (DSM) in 2010 and by Kraft Foods for 2011 and 2012. These sponsors however did not have any role in the planning, design or conduct of the course. The USAID (United States Agency for International Development) -funded Global Nutrition Collaborative Research Support Program (Nutrition CRSP) supported the travel, accommodation and food expenses for 6 students from Nepal and one from Uganda in 2012. Funding for the rest of course expenses (international airfare, accommodations, textbook purchases for SJRI library, local travel) were supported by the core BBNC grant from a Boston-based foundation.

#### Facilities and infrastructure at SJRI

The St. John’s Research Institute was established in 2004 with the vision to improve the health of the community and patients through research and the development of a centre of excellence in medical research in India. The BBNC short courses are conducted at SJRI using the facilities and infrastructure available, which include a state of art 220 seat auditorium, where the didactic sessions are conducted in the morning. The air-conditioned auditorium is equipped with individually-controlled microphones and electrical outlets at each seat to optimize audience participation, and possesses excellent acoustics. SJRI also has fully equipped body composition, molecular biology, and biochemical laboratories that are used to give the students practical hands-on experiences. A BBNC library was started in 2010, which contains textbooks on nutritional epidemiology, biostatistics, physiology, molecular nutrition, and human nutrition [11-15]. Each year, books are added to the library and the students make use of the library during the two weeks of the course. A video-conference system was funded by the BBNC collaboration to support lectures by faculty from Boston. Students in need of laptop computers were provided with these for the course duration and for the hands-on data analysis sessions.

#### Course content and format

The central component of the BBNC was to develop new interdisciplinary curricula in nutrition research methodology. The BBNC short course was planned with didactic lectures in the morning, supplemented with interactive, problem-based discussion and practical hands-on sessions in the afternoons. This course introduced students to nutrition research methods, through critical evaluation of the scientific literature and exploration of a variety of biological, epidemiological, demographic, and social factors that affect nutritional status.

The course was structured into substantive, methodological, application, case study and hands-on practical sessions. Sessions were jointly designed and implemented by faculty members from India and Boston. Case studies were used to explore ethical issues in nutrition research and programs. Table 1 lists the topics covered during the short course. Adequate time was provided for lunch and tea breaks, which encouraged the students and faculty to become acquainted and discuss mutual research interests in an informal environment. Since the faculties were experts in their fields at both national and international levels, a session was held where all faculty from three institutions led a roundtable discussion on career development in the field of nutrition research. Different perspectives from India and US-based institutions were explored and potential opportunities and barriers were discussed.

**Table 1:** List of topics covered during the Bangalore Boston Nutrition Collaborative short course in Nutrition Research Methodology (2010–2012)

**Substantive**	**Practical**
○ Nutritional physiology	○ Nutritional assessment
○ Nutritional Biochemistry	○ Extracting evidence from internet
○ Molecular nutrition	○ Body composition
○ Nutrition and global health	○ Laboratory methods in nutrition research
○ Ethics in nutrition and global health	○ Novel diagnostics
○ Health policy and nutrition	○ Biostatistics
○ Nutrition and food security in emergencies	○ Survey design & data collection
○ Waterborne diseases and public health	○ Data analysis
○ Childhood obesity and adult outcomes	
○ Nutrition and perinatal health	
**Methods**	**Case studies/journal club**
○ Intervention study design	○ Nutrition and infection
○ Epidemiological research methods	○ Estimation of amino acid requirements
○ Nutritional epidemiology and surveillance	○ Ethics of nutritional interventions
○ Infectious disease epidemiology	○ Clinical nutrition
○ Obesity and chronic disease epidemiology	○ Research in nutrition and perinatal health
○ Scientific manuscript development	○ Nutrition in crisis zones
○ Nutritional assessment	○ Nutrition and epidemiologic transitions
○ Micronutrients and stable isotopes	○ Nutrition in emergencies
○ Statistical techniques for nutritional studies	
○ Programmatic research in nutrition and perinatal health	
○ Nutrigenetics and vitamin A	
○ Physical activity interventions	
Practical aspects of running a clinical trial	

All the sessions, including the hands-on sessions, were videotaped and are currently being compiled to be uploaded onto a web learning module. Each student at the end of the two- week course received a course completion certificate signed by all the course directors of the three institutions, a BBNC memento, a group photograph and an alumnus pin.

#### *Research proposal*

In addition to the core curriculum, students explored specific research areas through the development of a research proposal, with mentorship from the faculty members. Students were assigned into teams with colleagues from different institutions in groups of three or four to develop a one-page synopsis and a 20-minute oral presentation on their proposed nutrition research project. The research projects were in the form of either 1) an intervention to target a nutritional problem, 2) laboratory investigation to examine a nutritional problem, 3) formative research to examine a nutritional problem, or 4) a topic of their choice in nutrition research. Proposals included background/rationale, research methods including power calculations and statistical analysis plans, anticipated results, and budget. Students presented their research proposals to a panel of faculty members, who independently evaluated the research proposals based on the abstract, presentation, and the post-presentation discussion. The winning team received a small cash award and a special certificate. This practical exercise gave the students an experience of proposal development, planning and teamwork.

#### BBNC alumni website

An alumni website was established after the first course. This website was developed using a social network framework (http://www.ning.com) and provides a platform for the students to continue their interaction with the faculty and each other after the course. The website has different features of a social network platform such as blog posts, discussion forum, online chat, moderated content upload including videos, pictures and text. The website also has a message broadcast feature to send mass emails to all group members. A notice board for events is available. A total of 95 members including 14 faculty have registered on this platform. Although the website is rich in features, the utilization of this website has not been optimal by the alumni. Newer strategies to improve interaction and communication using this website are being planned.

### II. Web-based learning system

Internet- or web-based learning approaches are becoming increasingly utilized in medical and other graduate education, and there is evidence suggesting that on-line learning is equally effective [16-20] or perhaps even more effective than classroom learning [21]. One of the aims of the BBNC was to setup a web-based learning system that could also be used for distance learning. The specific objectives were to develop distance-learning curricula involving lectures, web-based case discussions and shared curricula. To this effect, Tufts University offered their tested E-learning platform called Tufts University Sciences Knowledgebase (TUSK) and the implementation of this platform was supported by funds from the BBNC grant. St. John’s supported the hardware and networking costs for the implementation of this application.

### Learning management system – TUSK

Tufts University Sciences Knowledgebase (TUSK) is a vast learning management system developed by Tufts University [22]. It enables faculty to upload teaching materials such as slides, documents, videos, pictures and sound files for students to access. This TUSK system has been modified and adapted as ‘TYRO’ for St. John’s National Academy of Health Sciences and uses the entire learning management system’s engine developed by Tufts University. The application has a course management system similar to what is available on commercial systems. However, this system has far greater capability in enabling faculty and students to link to information related to a particular topic across all courses. It also has a feature where existing content can be reused in new contexts. The other features of this platform include display of schedules, on-line quizzes, discussion boards, notifications and evaluations. It also facilitates indexing using the National Library of Medicine’s Unified Medical Language System.

**Figure 1:**
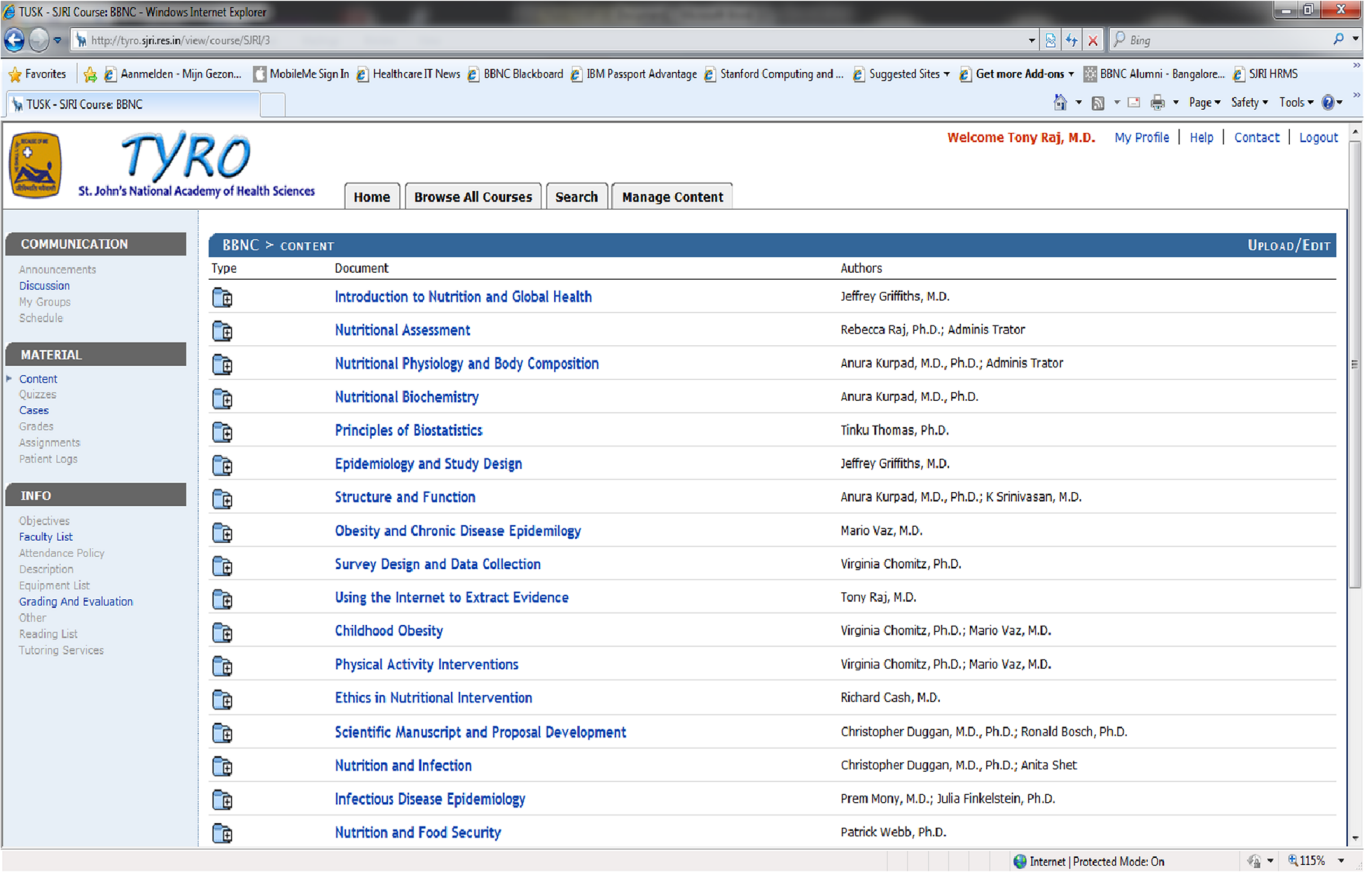
Screen shot of the Web Learning system (TYRO) used in the Bangalore Boston Nutrition Collaborative short course on Nutrition Research Methods.

The digital repository for the BBNC has been carefully designed based on the course curricula that has been taught from 2010–2012. The course content for the BBNC has been uploaded onto TYRO based on topics. Students can login using secure individual login accounts and can access lecture materials such as slides, pictures and other reading material online. Students have the ability to make their own notes and flash cards online for each lecture. In the future, more content such as videos, cases and pre- and post-testing will be added. Figure 1 depicts a screen capture of the BBNC course on TYRO. This learning management system will also augment the BBNC short course by enabling students to continue participation outside of the classroom and stimulate further engagement related to nutrition research.

### III. Graduate training opportunities at HSPH and tufts

The third goal of the Bangalore Boston Nutrition Collaborative was to provide opportunities for training in Boston for Indian students or junior faculty. The Harvard School of Public Health and Tufts University have internationally recognized graduate degree programs in the fields of nutrition, epidemiology, and biostatistics.

## Results

### Student application

During the first year of the course (2010), 27 students were selected from 153 applications across India. In the second year of the course, 27 students were selected from 98 applications across India, South Asia, and Sub-Saharan Africa. In 2012, 35 students were selected from 79 applicants from India, Nepal, Pakistan, Bangladesh and Uganda. In the first year, information about the course was sent to almost all health-related graduate and post graduate colleges, government and non-government institutions in India. Subsequently, we felt that PhD scholars and those actively involved in research would most benefit from the course. We therefore in the subsequent years, sent out the information only to PhD scholars and those actively involved in research in academic, governmental and non-governmental institutions. The narrowing of this target group may be one reason for the number of applications to have decreased from the first year. Applicants included medical students, nurses, physicians, dieticians, and allied health professionals who were involved in clinical, research, program, policy, or laboratory work as well as junior faculty from medical, nursing, and public health institutions. Details of the participants who were selected for the three courses are provided in Table 2.

**Table 2:** Characteristics of students/junior faculty participating in the Bangalore Boston Nutrition Collaborative short course in Nutrition Research Methodology (2010–2012)

	**2010**	**2011**	**2012**
**Number**	**27**	**27**	**35**
**Gender**			
• Female	24 (89%)	22 (81%)	25 (71%)
**Education level**			
• Ph D	1 (4%)	8 (30%)	4 (11%)
• Pursuing Ph D	4 (15%)	9 (33%)	10 (29%)
• Post graduate (Medical)	1 (4%)	4 (15%)	2 (6%)
• Post graduate (Non -medical)	18 (67%)	6 (22%)	17 (49%)
• Graduate	3 (11%)		2 (6%)
**Institution**			
• Government organization/hospital	1 (4%)	2 (7%)	10 (29%)
• Academic government	4 (15%)	5 (19%)	6 (17%)
• Academic private	11 (41%)	7 (26%)	14 (52%)
• Private hospital/research/institute	11 (37%)	13 (48%)	5 (14%)
**Geographic distribution**			
• South India	13 (48%)	12 (44%)	9 (26%)
• North India	14(52%)	13 (52%)	16 (46%)
• Outside India		2 (7%)	10 (29%)

Values in parenthesis are percentage.

### Faculty and staff

BBNC short course faculty has included 26 members: St. John’s Research Institute (16), Harvard University (4), Tufts University (4), Cornell University (1) and Public Health Foundation of India (1). Faculty were selected based on their areas of subject expertise and teaching skills. Faculty members were 8 (32%) female, and represented a wide range of academic appointments, with 7 (28%) Professors, 10 (40%) Associate Professors, 5 (25%) Assistant Professors, and 3 (7%) Lecturers, Research Scientist or other positions. Faculty were drawn from the fields of clinical nutrition, public health, epidemiology, biostatistics, information technology, physiology, health policy, pediatrics, and molecular biology.

### Course evaluation and feedback

Individual sessions were evaluated by students, with criteria on a 5-point Likert scale, and an open-ended section for feedback on the course. Criteria such as overall organization, presentation of ideas and concepts, engagement of audience in discussions, relevance of readings were used to evaluate each session. A detailed evaluation of the course was conducted with questionnaire and interviews with participants and faculty members. The quantitative survey consisted of 16 closed-ended and six open-ended questions, pertaining to the course structure, content, design, and implementation. Students reported their rating of the overall course experience, with reference to an ideal course in their perception (maximum score of 100) as a percentage. Ratings on the design (9 questions –whether course met expectations, sessions were well planned, new knowledge was gained, application session helped in the understanding of course, the use of examples were adequate, level of course was appropriate, textbook was useful, case studies and practical sessions helped to understanding the course and the course would be recommended to others) and implementation (4 questions- clarity of lecture, effectiveness of teaching aids, adequacy of course material, interaction of faculty with students) were obtained on a 5 point Likert scale ranging from “Strongly disagree to Strongly agree”. Subsequently, a total design score and an implementation score for each student were obtained and converted to a percentage of the maximum possible total score under each category. The overall course rating, design and implementation scores were compared using Chi-square test across the three years by categorizing the percentage score as “>90%, 81-90%, 71-80%, 61-70%, 51-60% and 41-50%”. None of the students rated below 40% during any of the years. The feedback was not compulsory but the students were encouraged to complete the process and all the students filled in the forms. The feedback was confidential and the students did not report their name/institution. Very minor changes were made in the feedback form after year 1 (in 2011) to improve the clarity and this was taken into consideration during the pooling and analysis of data. The feedback was shared with the faculty after the course.

Qualitative entrance and exit interviews with each participant were conducted to note their experiences during the course, recommendations for improvement, what they learned, and how they planned to apply their training in the course to their future nutrition research. Selected faculty members were also interviewed regarding their experiences leading sessions and working with participants in the course.

**Figure 2:**
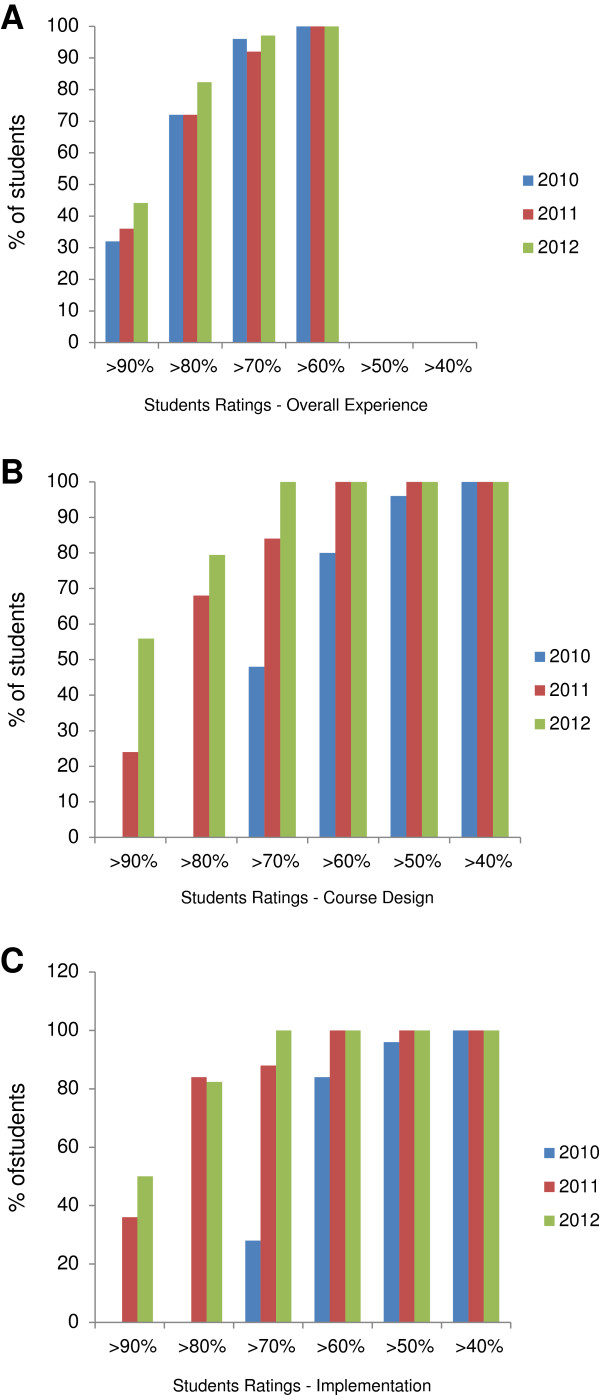
Student ratings each year 2010–12 for different aspects of the course: A) Overall course experience (Comparison between years, Chi-square p = 0.94), B) Course design (p < 0.001), and C) Course implementation (p < 0.001).

In general, student feedback about the course was excellent. Figure 2 depicts the comparative feedback from students, across the three years (2010 – 2012) on three metrics: overall course rating, course design, and course implementation. The bars represent the percentage of students who rated above a given percentage score.

The rating of the overall course experience (Figure 2A) was excellent and consistent across the years. In 2010 and 2011, 19 (72%) of students rated the overall course experience as higher than 80% , increasing slightly to 29 (82%) in 2012 (p = 0.94 by chi squared test). Ratings for course design (Figure 2B) and implementation (Figure 2C) improved significantly over the years. In 2010, none of students rated the design of the course higher than 80%, whereas in 2011 and 2012, 17 (63%) and 28 (80%) respectively gave this rating (p < 0.001 by chi squared test). There was a similar increase in students who rated the implementation of the course at higher than 80%, from nil in 2010 to 23 (84%) and 29 (83%) in 2011 and 2012 respectively (p < 0.001 by chi squared test).

Open-ended questions were also asked on the strengths, weaknesses and suggestions for the course. The opportunity for our Indian students to learn with and from students from Pakistan, Bangladesh, Nepal, Uganda and Cambodia was invaluable and greatly enhanced the learning experience. The students also reported that the nutrition research project presentation provided them an opportunity and confidence to gather knowledge from the existing literature, work as a team and make presentations to an audience. A list of some of the projects presented by the students is provided in Table 3. The hands-on sessions were the highlight of the course, receiving the maximum mention by the students and providing an opportunity to the students to apply the knowledge gained in the substantive and methods sessions. Some of the suggestions from the students were to increase course duration and frequency, plan an advanced course, make the course less hectic, and to expand the curriculum to include topics such as food security, geriatric nutrition and child nutrition. Strengths of the course reported by the students through open ended questionnaires:

1. "Excellent faculty with interactive teaching, good use of audio visual aids".

2. "Faculty were Par Excellence".

3. "Very well organized course, every query, concern and needs were addressed".

4. "Excellent practical hand on experience".

5. "Comprehensive, interdisciplinary course content".

**Table 3:** List of Student Research Proposals

	**2010**	**2011**	**2012**
1	Nutritional Status of Children diagnosed with Autism as compared to Normal children	Development and validation of a Photographic Food Atlas of selected Indian Foods for quantifying portion size among adults	Impact of zinc supplementation on immune response to oral polio vaccine: A double blind, randomized controlled trial
2	Effect of dietary intervention of monounsaturated fatty acid in subjects with metabolic syndrome	Prevalence of organophosphate toxicity in farmers of South India	Impact of Built environment on obesity & physical activity patterns among children in affluent schools of Delhi
3	Prevalence of impaired fasting glucose and nutritional intervention	Prevalence of cardiovascular disease in hypercholesterolemic type 2 diabetic: Role of Psyllium husk and lifestyle intervention	Food Security among households of a district in India – Impact of Food Subsidization on Nutritional Profile of Adolescent Girls
4	Study of the prevalence of Anemia and Micronutrient deficiencies among pregnant south Indian women and possible effects on their Birth outcome	Formulation of hypoglycemic breakfast foods and to evaluate their suitability in improving glycemic status among young adults with pre diabetes - a multicentric evaluation	Maternal Determinants Of Body Composition of Newborn
5	A comparative study to assess the growth of the infants and Feeding practices among HIV infected and non infected mothers in Bangalore	Impact of combined supplementation of phytosterols and omega 3 fatty acids in subjects with metabolic syndrome	Efficacy of vitamin D supplementation on incidence of acute lower respiratory infection among young children in Sarlahi, Nepal
6	Effect of supplementation of ω-3 fatty acid, EPA, DHA on ADHD children	Impact of intensive nutritional lifestyle intervention during pregnancy on infant birth weight: a pilot study	Impact Of Low Maternal Vitamin D Levels (Hypovitaminosis D) During Gestation On Newborns
7	Facility provision in Primary schools correlates with physical activity and childhood obesity	Efficacy of Probiotic in 6–69 months of severely malnourished children in India	Effects of MNP (Micro Nutrient Powder) Sprinkles vs IFA tablet supplementation during pregnancy & postpartum, on maternal nutritional status, birth outcome and child growth.
8	A comparative study to assess the growth of the infants and Feeding practices among HIV infected and non-infected mothers in Bangalore	Impact of Nutrition Intervention during Lactation on Breast Milk composition and offspring growth and development	Assessment of Calcium and Vitamin D levels in association to obesity among school going children

### Alumni survey and feedback

In order to track the BBNC alumni students, we conducted an electronic mail survey in February 2012. Student alumni from 2010 and 2011 were invited to complete the survey. Of the fifty four students, two were not able to be contacted by telephone, mail or email. Fifty two of the alumni students from the 2010 and 2011 courses were contacted through the BBNC alumni site, email and phone calls. Thirty-seven students (71%) responded and completed the survey. The information collected from the survey included present position, current employer, number of proposals submitted for funding, number of manuscripts written, and whether the BBNC course helped them professionally and met its objectives. Of the 52 students surveyed, 14 (37%) were employed in academic medical institutions while 11 (29%) were employed in governmental or nongovernmental institutions, with the remaining in industry. Twenty-eight (76%) were actively involved in research, 20 (54%) had published manuscripts, and 13 (34%) had submitted proposals for external funding. Thirty-four (93%) agreed that the BBNC course met its objectives and helped them professionally.

### Faculty and student exchange

With support from BBNC funds, 3 faculty visited HSPH and Tufts for collaborative research efforts, and a PhD student visited HSPH for data analysis and writing of a research publication [23]. Two more students visited HSPH for data analysis and writing of a research publication in 2013. An alumnus from the 2010 BBNC course was also selected for doctoral studies in Nutrition at HSPH. The selection criteria for training opportunities in Boston included interest and involvement during the short course, educational background, commitment to nutrition and potential to transfer knowledge back to the parent institution.

## Discussion

The Bangalore Boston Nutrition Collaborative was designed to build capacity and to provide research training for young professionals in the fields of nutrition and global health from India and subsequently other countries in the region. This international course in nutrition research methods, conducted annually since 2010, has provided 89 students with a unique global health education experience and an opportunity to explore new approaches and strategies to solve nutrition-based public health problems including anemia, undernutrition, stunting, cardiovascular disease, obesity and diabetes. Research projects helped stimulate the students to work together in related fields and topics, thus facilitating future partnerships with institutions around the world. Survey results from our alumni students show that a majority (76%) of the students are presently involved in research, and that more than 50% have published manuscripts after the course. Ninety three percent of the alumni agreed that the course helped their career. The alumni association and TYRO system may provide the nucleus of an informal international network to promote nutrition research.

The 12th five-year plan (2012–2017) of the Government of India focuses on restructuring public health schemes and plans to raise the allocation of funds for health care from the current 1.1% of gross domestic product to 2–2.5% [24]. The planning commission recognised that there is acute shortage of trained health personnel (medical and non -medical) and attributed this to be one of the main reasons for the inadequate provision of health services in India. They emphasised that public health education courses should be multidisciplinary, problem solving and open for both medical and non medical persons [7]. Since nutrition is a key component of public health, proper training of professionals is crucial in setting up good health systems, and evaluating the success of their programs.

Khandelwal et al. 2011 conducted a situational analysis to map the existing educational initiatives for nutrition and public health nutrition in India [9]. They concluded that India gravely lacks dedicated education and training programmes in PHN. Nearly 50% of the available nutrition courses were noted to focus on Food Science and Technology [9]. An inspection of syllabi (through internet and available material) offered in some of these courses shows that they are mainly theoretical. In order to provide practical experience in nutritional methods, colleges would have to be well equipped with laboratories having instruments to demonstrate the practical difficulties in measuring, for example, body composition, nutritional physiology and biochemistry and problems in clinical nutrition. The investment to provide a course with such hands-on experience is very large in terms of infrastructure, equipment and expertise and these costs make it prohibitive for the colleges to be able to provide such a practical training to their students. Short courses help in training of specific skills and methodologies and can be more responsive to changing nutritional needs and problems [9]. Institutions in India such as the Public Health Foundation of India (PHFI), National Institute of Nutrition (NIN), Nutrition Society of India, Nutrition Foundation of India and International Life Sciences Institute offer short training courses in research, nutrition and public health [9]. The BBNC short course however, was planned as an intensive interdisciplinary nutrition research methodology course, aimed to train the students in planning research studies from “Cell to Society”, with adequate practical sessions, and with an optimal student–teacher-instrument ratio, so that the “hands-on” principle was adhered to.

The main strength of our short course was the “excellent faculty” from SJRI and the faculty from Boston. However, we understand that we will need further funding to sustain the involvement of the international faculty and efforts are ongoing to obtain funding from governmental and non-governmental agencies. Future education efforts of BBNC will include expansion of graduate student short courses and mentored research experience in Boston, addition of faculty exchanges to promote collaborative education and research projects, and joint development of courses to be taught in both the short course format at SJRI and/or hosted on the BBNC website. Additionally, we are hoping to set up the system by which faculty who cannot travel to India can give their sessions through video conferencing system. Assignments for the future short courses, as well as student and faculty evaluations, will be performed using the learning management system TYRO. The BBNC short course may be extended to other regions in South Asia, Africa and the world as part of the distance education objective and thus hopefully will set a standard in high-quality nutrition education using a collaborative approach enabled by information and communication technologies.

## Conclusions

The BBNC program has attracted students from different backgrounds and offered transformative opportunities for them to be collaborative leaders of future global health research, relying on a short-term course in research methods, the opportunity for distance learning, and research experience in Boston. The lessons learned from this international course could provide valuable information for future courses in other institutions. This course can be seen as a success based on positive evaluations by the students. The quality of speakers, noted to be the main strength, could be attributed to the use of speakers from multiple institutions. The short course of BBNC provided the students with hands-on experience of working closely with international and national experts to gain in-depth knowledge of nutrition research methodology skills and learn systematic methodic research and teaching skills which are urgently required to strengthen the public health cadre in India. In addition, we feel that our collaborative efforts can take advantage of the existing strengths and expertise of the various programs, resources and research of the three institutions and result in greater credibility and productivity in the discipline of nutrition and global health education. Sustainability of the collaboration, faculty and institutional support is crucial to ongoing success and we are planning to apply for funding which will help in conducting courses for the next 5 years. We have recently received an award from the United States India Education Foundation - Obama Singh 21st Century Knowledge Initiative. One of the main goals of this initiative is to conduct the short course in nutrition research methodology and offer the course to students without any course fees. As a part of this program, students and faculty from St. John’s and Harvard will participate in mutually beneficial exchange visits for three years, with clear educational and research objectives in field of nutrition and public health.

## Abbreviations

BBNC: Bangalore Boston Nutrition Collaborative; SJRI: St. John’s Research Institute; HSPH: Harvard School of Public Health; DSM: Dutch States Mines; USAID: United States Agency for International Development; CRSP: Collaborative Research Support Program; TUSK: Tufts University Sciences Knowledge base.

## Competing interests

The authors declare that they have no competing interest.

## Authors’ contributions

RK, JLF, JKG, RB, AVK and CD contributed to the conceptualization, design and implementation of the course. All the authors were involved in writing the manuscript. TT and RB were involved design of the biostatistics components of the course. TR designed the alumni website and web based learning system. JKG, AVK and CD secured funding for BBNC. All authors read and approved the final manuscript.

## Pre-publication history

The pre-publication history for this paper can be accessed here:

http://www.biomedcentral.com/1472-6920/14/5/prepub
